# Wandering Ozurdex^®^ implant

**DOI:** 10.1007/s12348-011-0042-x

**Published:** 2011-09-30

**Authors:** Reema Bansal, Pooja Bansal, Pandurang Kulkarni, Vishali Gupta, Aman Sharma, Amod Gupta

**Affiliations:** 1 Advanced Eye Center, Post Graduate Institute of Medical Education and Research, Chandigarh, India; 2 Department of Internal Medicine, Post Graduate Institute of Medical Education and Research, Chandigarh, India; 3 Department of Ophthalmology, Advanced Eye Centre, Post Graduate Institute of Medical Education and Research, Chandigarh, 160012 India

**Keywords:** Aphakia, Intravitreal injection, Ozurdex^®^, Uveitis, Lensectomy, Vitrectomy

## Abstract

**Purpose:**

To report the behavior of intravitreal Ozurdex^®^ implant in eyes with post-lensectomy–vitrectomy (PLV) aphakia.

**Methods:**

Retrospective chart review of three eyes with PLV aphakia (three patients with uveitis) who received intravitreal injection of Ozurdex^®^ for cystoid macular edema (one eye), persistent inflammation (one eye), and ocular hypotony (one eye). Final outcome was assessed in terms of effectiveness, stability, and tolerance of the implant.

**Results:**

Following the implant, an initial improvement was seen in all the three eyes. However, the implant migrated into the anterior chamber (AC) at 1 week in two eyes and at 5 weeks in one eye, and wandered between the AC and vitreous cavity with changing postures of the patient. Two eyes developed corneal edema, of which one eye underwent implant removal from the AC.

**Conclusion:**

Ozurdex^®^ implant should be contraindicated in eyes with PLV aphakia to avoid its deleterious effect on the corneal endothelium.

Corticosteroids have been the mainstay for treatment of noninfectious uveitis. They can be administered systemically or locally by topical, periocular, or intravitreal routes. Persistent inflammation and cystoid macular edema (CME) secondary to ocular inflammation are often vision-threatening and pose a significant therapeutic challenge.

The Ozurdex^®^ (Ozurdex^®^; Allergan, Inc, Irvine, CA, USA) dexamethasone drug delivery system (DDS) is a biodegradable intravitreal implant that delivers sustained release of 700 μg of preservative-free dexamethasone to the retina and vitreous. It is approved by the United States Food and Drug Administration as a first-line therapy for the treatment of macular edema following branch or central retinal vein occlusion, as well as for noninfectious posterior uveitis [1–3].

The results of its use in uveitis in clinical practice and animal models for controlling inflammation and improving CME and visual acuity have been encouraging [4, 5]. However, it has not been studied in aphakic eyes with uveitis so far. We report our experience with the intravitreal use of Ozurdex^®^ implant in three eyes (three patients) with post-lensectomy–vitrectomy (PLV) aphakia.

## Methods

We did a retrospective chart review of three patients with uveitis who received intravitreal Ozurdex^®^ in eyes with PLV aphakia. Demographic features, location and etiology of uveitis, systemic therapy and surgical intervention prior to Ozurdex^®^ injection, indications for the injection, best corrected visual acuity (BCVA) and intraocular pressure (IOP) before and after injection, and final outcome in terms of effect, stability, and tolerance of the implant were noted. Ozurdex^®^ was injected into the vitreous cavity under sterile conditions in accordance with the manufacturer’s instructions.

## Results

All three patients were males (Tables 1 and 2). All three aphakic eyes had undergone pars plana lensectomy (PPL) and pars plana vitrectomy (PPV) for visually disabling cataract due to chronic uveitis. The indications for Ozurdex^®^ injection were CME (one eye), persistent intraocular inflammation (one eye), and ocular hypotony (one eye). An initial improvement was seen in all the three eyes post-injection. However, the implant migrated into the anterior chamber (AC) at 1 week (two eyes) and 5 weeks (one eye) post-injection and wandered between the AC and vitreous cavity with changing postures of the patient. Further follow-up revealed its adverse effect on the corneal endothelium in two eyes till the last visit, compromising visual acuity and IOP.

**Table 1:** Baseline characteristics and behavior of intravitreal Ozurdex implant in post-lensectomy–vitrectomy aphakic eyes

Case	Age	Sex	Diagnosis	Eye	Pre-implant BCVA	Pre-implant IOP (mmHg)	Indication for Ozurdex implant	Treatment	Interval between implantation and migration into AC
1	47	M	Chronic anterior uveitis	Right	6/24	15	Cystoid macular edema	Oral CS + azathioprine	1 week
2	13	M	Behcets disease	Right	Counting fingers	14	Persistent intraocular inflammation	Oral CS + azathioprine/infliximab/cyclosporine	8 days
3	15	M	Chronic anterior uveitis	Right	6/36	6	Ocular hypotony	Topical anti-inflammatory/cycloplegic therapy	5 weeks

*M* male, *BCVA* best corrected visual acuity, *IOP* intraocular pressure, *CS* corticosteroids, *AC* anterior chamber

**Table 2:** Outcome of intravitreal Ozurdex implant in post-lensectomy–vitrectomy aphakic eyes

Adverse effects of migrated implant	Surgical removal of Ozurdex implant	Final outcome	Post-implant BCVA	Post-implant IOP (mmHg)	Follow-up after implant migration, months	Final location of the implant
Corneal edema	No	Resolution of cystoid macular edema; resolution of corneal edema	6/18	12	4	Vitreous cavity
Corneal edema, elevation of IOP	Yes, 40 days after implantation	Regression of inflammation, control of IOP, corneal edema persisted	6/18	33	4	Removed from the eye
None	No	Improvement of hypotony	6/6	12	3	Vitreous cavity

## Case reports

### Case 1

**Fig. 1:**
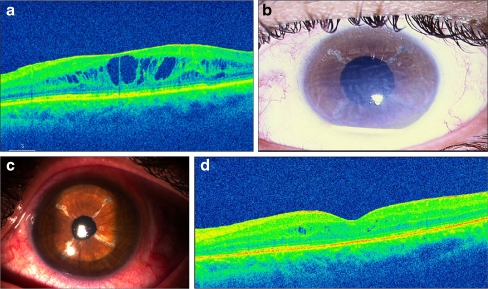
**a** Optical coherence tomography (OCT) of the left eye of a 47-year-old male (case 1) showing cystoid macular edema (CME) 8 weeks following pars plana lensectomy and vitrectomy for significant cataract due to chronic anterior uveitis. **b** Slit lamp photograph of anterior segment of the same eye as in (**a)**, 1 week after the intravitreal implantation of Ozurdex^®^, showing migration of the implant into the anterior chamber with corneal edema. **c** Slit lamp photograph of the same eye as in (**a**, **b)**, showing clear cornea 4 months after Ozurdex migration. **d** Optical coherence tomography (OCT) of the same eye as in **a**–**c**, showing insignificant intraretinal cystic spaces 3 months after intravitreal implantation of Ozurdex^®^

A 47-year-old male was referred to our clinic with bilateral chronic anterior uveitis of 5 years duration. The BCVA was counting fingers in both eyes and IOP 15 mmHg and 10 mmHg in the right and left eye, respectively. The anterior segment showed 1+ cells and 1+ flare, with multiple posterior synechiae and significant cataract in both eyes. The posterior segment was unremarkable. Topical anti-inflammatory and cycloplegic therapy was started, along with oral corticosteroids and immunosuppressive therapy (azathioprine, 2 mg/kg/day). At 8 months of follow-up in our clinic, he underwent PPL and PPV in the left eye. At 8 weeks postoperatively, the left eye had BCVA 6/24, IOP 15 mmHg and significant CME (Fig. 1a). Despite systemic and local therapy, the CME persisted in this eye at 4 months with BCVA 6/24 and IOP 15 mmHg. He was given Ozurdex^®^ injection. At 1 week post-injection, the CME resolved although the BCVA remained 6/24. The IOP was 11 mmHg. However, the Ozurdex^®^ implant migrated into the AC, and there were descemet’s folds with corneal edema (Fig. 1b). At 18 days post-injection, the BCVA was 6/24, IOP 16 mmHg; cornea was clear; there was no CME, and the implant was back in the vitreous cavity. The patient was cautioned and instructed to avoid prone position. On his last visit, at 4 months post-injection, the BCVA was 6/24, IOP 12 mmHg, cornea was clear, and the implant was in the vitreous cavity (Fig. 1c). The OCT showed few intraretinal cystic spaces (Fig. 1d).

### Case 2

**Fig. 2:**
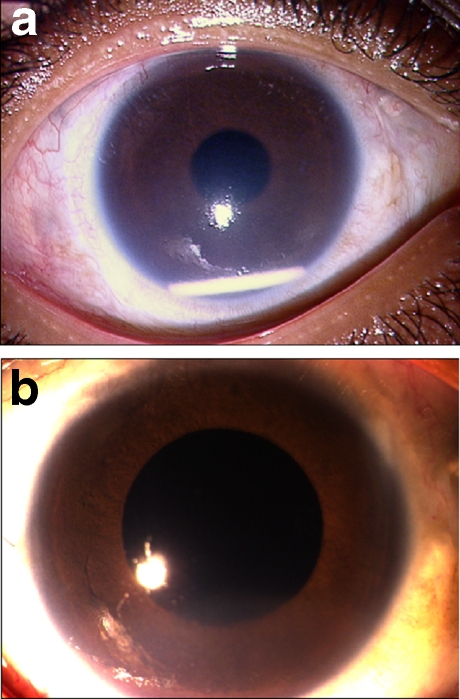
**a** Slit lamp photograph of the anterior segment of the right eye (following pars plana lensectomy and vitrectomy for complicated cataract) in a 13-year-old male with Behcets disease(case 2) showing migration of the Ozurdex^®^ implant into the anterior chamber and corneal edema, 14 days after intravitreal injection of Ozurdex^®^. **b** Same eye as in (**a**), 3 months after the removal of Ozurdex^®^ implant, showing persistent corneal edema, despite surgical removal of the implant

A 13-year-old male with Behcets disease presented to our center with bilateral panuveitis involving vasculitis and retinitis, treated elsewhere for 4 years. Despite sequential immunosuppressive therapy with azathioprine, infliximab, and cyclosporine, he had persistent vasculitis and retinitis in both eyes. The BCVA was counting fingers in both eyes with significant cataract. The IOP was 14 mmHg and 16 mmHg in the right and left eye, respectively. At 17 months of follow-up in our clinic, he underwent PPL and PPV in the right eye for complicated cataract. At 4 weeks postoperative, the BCVA improved to 6/18. However, new retinitis lesions appeared in the right eye. The IOP was 10 mmHg. He was given intravitreal Ozurdex^®^. Eight days post-injection, the BCVA was 6/18 and IOP 18 mmHg. The retinitis lesions regressed significantly. However, the Ozurdex^®^ implant had migrated into the AC. Cornea was clear. On the 14^th^ post-injection day, the BCVA was 6/18 and IOP was 18 mmHg. The implant was in the AC, and there was significant corneal edema in the lower half (Fig. 2a). Repositioning of the patient was attempted several times, but the implant would come back into the AC frequently. The IOP recorded on 18^th^ post-injection day was 28 mmHg. Topical dorzolamide hydrochloride 2% and timolol maleate 0.5% eyedrops were started. Despite combination anti-glaucoma therapy for 17 days, the IOP rose to 33 mmHg on 35^th^ post-injection day. Because of persistent corneal edema and raised IOP, the implant was surgically removed from the AC. Three months following its removal, the BCVA was 6/24 and IOP was 17 mmHg. Diffuse corneal edema decreased (Fig. 2b) but persisted, and the posterior segment was quiescent.

### Case 3

**Fig. 3:**
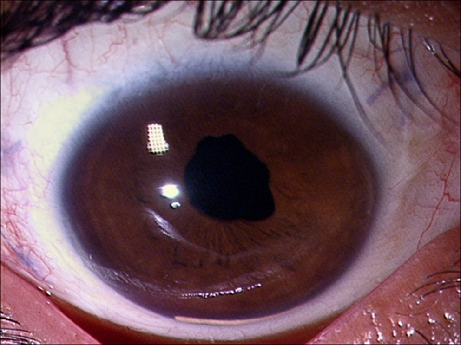
Slit lamp photograph of anterior segment of the right eye, following pars plana lensectomy and vitrectomy with SF_6_ tamponade with intravitreal injection of Ozurdex^®^ for complicated cataract, in a 15-year-old male (case 3) showing migration of implant into the AC, 5 weeks after the surgery

A 15-year-old male with idiopathic chronic anterior uveitis in the right eye since 4 years had BCVA 6/36 and IOP 6 mmHg. The left eye was normal. He had significant cataract in the right eye and underwent PPL and PPV. Intraoperatively, atrophy of ciliary processes was noted along with multiple peripheral retinal breaks. An internal tamponade with SF_6_ was done along with intravitreal injection of Ozurdex^®^. At 3 weeks postoperative, the BCVA was 6/12 and IOP was 18 mmHg. The eye was quiet, and the Ozurdex^®^ implant was well placed in the vitreous cavity inferiorly. At 5 weeks, the implant was found lying inferiorly in the AC (Fig. 3). The BCVA was 6/12, and IOP was 8 mmHg. The cornea was clear. The implant migrated back into the vitreous cavity once the patient attained supine position. On his last visit at 4 months, the BCVA was 6/6, IOP 12 mmHg, and the eye was quiescent with the implant in the vitreous cavity.

## Discussion

Ozurdex^®^ has been reported to be well tolerated in uveitis as well as in retinal disorders. So far, the ocular adverse effects reported after intravitreal injection include cataract formation, IOP elevation, subconjunctival hemorrhage, hyperemia, and conjunctival edema. These are often temporary and can be medically managed.

In all our patients described above, there was an initial improvement following intravitreal Ozurdex^®^ injection, in terms of CME (one eye), control of inflammation (one eye), and hypotony (one eye). However, the retention of Ozurdex^®^ implant in the vitreous cavity became a major challenge as it wandered between the AC and the vitreous cavity.

The IOP elevation was temporary and was medically managed. Corneal edema could be medically managed in one eye but necessitated a repeat surgical intervention in the other eye for removal of the Ozurdex^®^ implant.

The encouraging role of Ozurdex^®^ is due to its potency, dose consistency, extended duration of action, and minimal adverse effects [4–8]. Additional advantages of the biodegradable DDS are that they do not need to be retrieved and can be administered repeatedly. Myung et al. reported successful control of inflammation and CME in their patients with uveitis [4]. No serious ocular or systemic adverse effects were noted during the mean follow-up (post-injection) time of 5.25 months. The only concern in their experience with the implant was a shorter durability of its effect (about 3 to 4 months) than the typical 6-month period reported in retinal vein occlusion [1].

While the efficacy of Ozurdex^®^ in uveitis has been proved in clinical trials [2, 3], clinical case series [4], and experimental animal studies [5], none of the studies have reported its use and outcome in aphakic eyes of patients with uveitis after PPL and PPV.

The use of Ozurdex^®^ implant in vitrectomized eyes with diabetic macular edema has been reported recently by Boyer et al [9]. Their patients were phakic/pseudophakic, and the implant was well tolerated with an acceptable safety profile. Moreover, the authors believed that vitrectomized eyes responded well to sustained drug delivery with an implant.

To the best of our knowledge, our series is the first to report the behavior of Ozurdex^®^ implant and its outcome in eyes with PLV aphakia. In aphakic–vitrectomized eyes, the implant is placed within the vitreous cavity actually filled with aqueous fluid and therefore has the potential to migrate back and forth with changing postures and minimal resistance leading to complications as seen in our patients. Such behavior of the implant in these eyes is not unexpected. Moreover, the anterior migration of the implant at 5 weeks in case 3 after absorption of the gas may reflect the buoyancy of the implant in aqueous fluid and tendency to shift. We believe that Ozurdex^®^ injection should be contraindicated in eyes with PLV aphakia, even in the presence of recalcitrant macular edema due to any cause, to avoid its deleterious effect on the corneal endothelium.

## References

[1] Haller JA, Bandello F, Belfort R Jr, et al. Ozurdex® GENEVA Study Group (2010). Randomized, sham-controlled trial of dexamethasone intravitreal implant in patients with macular edema due to retinal vein occlusion. Ophthalmology.

[2] Kuppermann BD, Blumenkranz MS, Haller JA (2007). Randomized controlled study of an intravitreous dexamethasone drug delivery system in patients with persistent macular edema. Arch Ophthalmol.

[3] Williams GA, Haller JA, Kuppermann BD (2009). Dexamethasone posterior-segment drug delivery system in the treatment of macular edema resulting from uveitis or Irvine-Gass syndrome. Am J Ophthalmol.

[4] Myung JS, Aaker GD, Kiss S (2010). Treatment of noninfectious posterior uveitis with dexamethasone intravitreal implant. Clin Ophthalmol.

[5] Ghosn CR, Li Y, Orilla WC, Lin T, Wheeler L, Burke JA et al. (2011) Treatment of experimental anterior and intermediate uveitis by a dexamethasone intravitreal implant. Invest Ophthalmol Vis Sci [in press]. 10.1167/iovs.10-5939 21273539

[6] Herrero-Vanrell R, Cardillo JA, Kuppermann BD (2011). Clinical applications of the sustained-release dexamethasone implant for treatment of macular edema. Clin Ophthalmol.

[7] Saraiya NV, Goldstein DA (2011). Dexamethasone for ocular inflammation. Expert Opin Pharmacother.

[8] London NJ, Chiang A, Haller JA (2011) The dexamethasone drug delivery system: indications and evidence. Adv Ther [in press]. 10.1007/s12325-011-0019-z 21494891

[9] Boyer DS, Faber D, Gupta S, Patel SS, Tabandeh H, Li XY (2011). Dexamethasone intravitreal implant for treatment of diabetic macular edema in vitrectomized patients. Retina.

